# Multi-center validation of Catquest-9SF visual function questionnaire in Ontario, Canada

**DOI:** 10.1371/journal.pone.0278863

**Published:** 2023-07-06

**Authors:** Anna Kabanovski, Bindra Shah, Chelsea D’Silva, Julia Ma, Simona C. Minotti, Jenny Qian, Wendy Hatch, Robert Reid, Varun Chaudhary, Sherif El-Defrawy, Iqbal Ike Ahmed, Matthew B. Schlenker

**Affiliations:** 1 Department of Ophthalmology and Vision Sciences, University of Toronto, Toronto, ON, Canada; 2 Institute for Better Health, Trillium Health Partners, Mississauga, ON, Canada; 3 Department of Statistics and Quantitative Methods, University of Milano-Bicocca, Milan, Italy; 4 Institute of Health Policy, Management, and Evaluation, University of Toronto, Toronto, ON, Canada; 5 Department of Eye Medicine and Surgery, Hamilton Health Sciences, Hamilton, ON, Canada; Saarland University, GERMANY

## Abstract

**Purpose:**

To investigate the psychometric performance and responsiveness of Catquest-9SF, a patient-reported questionnaire developed to evaluate visual function as related to daily tasks, in patients referred for cataract surgery in Ontario, Canada.

**Methods:**

This is a pooled analysis on prospective data collected for previous projects. Subjects were recruited from three tertiary care centers in Peel region, Hamilton, and Toronto, Ontario, Canada. Catquest-9SF was administered pre-operative and post-operatively to patients with cataract. Psychometric properties, including category threshold order, infit/outfit, precision, unidimensionality, targeting, and differential item functioning were tested using Rasch analysis with Winsteps software (v.4.4.4) for Catquest-9SF. Responsiveness of questionnaire scores to cataract surgery was assessed.

**Results:**

934 patients (mean age = 71.6, 492[52.7%] female) completed the pre- and post-operative Catquest-9SF questionnaire. Catquest-9SF had ordered response thresholds, adequate precision (person separation index = 2.01, person reliability = 0.80), and confirmed unidimensionality. The infit range was 0.75–1.29 and the outfit range was 0.74–1.51, with one item (‘satisfaction with vision’) misfitting (outfit value = 1.51). There was mistargeting of -1.07 in pre-operative scores and mistargeting of -2.43 in both pre- and post-operative scores, meaning that tasks were relatively easy for respondent ability. There was no adverse differential item functioning. There was a mean 1.47 logit improvement in Catquest-9SF scores after cataract surgery (p<0.001).

**Conclusion:**

Catquest-9SF is a psychometrically robust questionnaire for assessment of visual function in patients with cataract in Ontario, Canada. It is also responsive to clinical improvement after cataract surgery.

## 1 Introduction

The demand for cataract surgery is growing worldwide [1], creating a challenging task to accurately evaluate appropriateness for surgery and decide on waitlist prioritization. Traditionally, visual acuity was the main indicator of visual function. However, other factors, like brightness, contrast, and glare, also affect visual function and must be considered [2, 3]. Thus, there is a growing need for a tool to accurately evaluate patients’ self-reported visual function as related to daily life.

Several quality-of-life questionnaires were created to measure visual function, including Catquest, Activities of Daily Vision Scale, Visual Functioning-14, and others [4–7]. These questionnaires were developed using Classical Test Theory (CTT), which was recently shown to have significant limitations that can be overcome with a newer approach called Rasch analysis [8]. In CTT, values assigned to responses are added together, which may be inaccurate because steps along the continuum may be unequal [9]. In Rasch analysis, data are converted to a scale with equally sized steps, which allows addition of scores, more accurate comparisons, and use of parametric statistics [9, 10].

Catquest was developed in Sweden using CTT in 1997 [11]. In 2009, Rasch analysis was applied to Catquest creating a new 9-item version called Catquest-9SF [12]. As of 2020, Catquest-9SF has been culturally adapted and validated in at least 12 countries and 12 languages (Chinese, Danish, Dutch, French, German, Italian, Malay, Portuguese, Russian, Spanish, English, and Swedish) [12–28]. In all populations, Catquest-9SF proved to be a reliable instrument for measuring visual function and it is currently frequently used to assess the visual function and satisfaction of patients undergoing cataract surgery, particularly with multifocal or extended depth-of-focus lenses [29–31]. Researchers who designed the Catquest-9SF in Sweden recently demonstrated that the questionnaire retained its long-term stable psychometric properties (over a period of 11 years) [31]. It is short and was rated as a higher-quality tool compared to others due to its psychometric performance and high responsiveness to cataract surgery [10, 32].

Despite the need for a visual function questionnaire, there are barriers for implementation of such a tool in routine care. For example, there was heterogeneity in the psychometric results across validation studies, suggesting that questionnaire performance can be population specific. Thus, questionnaires must be validated in new populations before use. Our group previously performed a Rasch validation analysis for Catquest-9SF in one center in Peel Region, Ontario, with pre-operative scores only [17]. This was the first validation study in Canada, and the results demonstrated excellent performance. In the following study we assess whether Catquest-9SF is valid in patients with cataract across three centers in different regions in Ontario, using pre- and post-operative responses. We also assess responsiveness to clinical improvement after cataract surgery and examine the performance of shorter versions.

## 2 Methods

### 2.1 Questionnaire

Catquest-9SF contains 9 items [12], with 2 global assessment items(Ca,Cb) and 7 activity-specific items(C1-C7). Each item has four response options, with a raw score of 1 representing better visual function and 4 representing worse visual function. ‘Cannot decide’ is an option for all items, which was treated as missing data. The English translation of Catquest-9SF was used (S1 Fig).

### 2.2 Participants

A pooled analysis was performed on prospective data collected for previous projects at three sites in Ontario, Canada: Kensington Eye Institute (Toronto), Trillium Health Partners (Mississauga), and St. Joseph’s Healthcare Center (Hamilton). Catquest-9SF was administered pre- and post-operatively to patients. Before combining the data from different sites, a sensitivity analysis was performed to ensure the groups were similar in demographics (S1 Table). This study was approved by the Trillium Health Partners Research and Ethics Board (#984), Hamilton Research Ethics Board, and University of Toronto Research Ethics Board (#27054). Written consent was obtained from participants.

### 2.3 Rasch analysis

In Rasch analysis, a single linear scale, measured in log of the odds (logit) units, is created to measure the person’s ability to perform a task (visual function) and the level of difficulty of the task (item difficulty) [33]. A person with a better visual function, or a difficult item, falls on the negative side of the scale. Rasch analysis was performed for two datasets using Winsteps software (v.4.4.4). The first analysis (Analysis A) included only pre-operative scores. All respondents with available pre-operative visual acuity for both eyes and no more than two missing responses on the pre-operative Catquest-9SF were included. The second analysis (Analysis B) was performed on stacked pre-operative and post-operative scores. Respondents from Analysis A with no more than two missing responses on the post-operative Catquest-9SF were included. A detailed explanation of the psychometric properties assessed was previously published [17]. Briefly, *category threshold order* shows whether response options are ordered appropriately, *infit and outfit* indicate if the data match the Rasch model, and *unidimensionality* determines whether the questionnaire only measures one trait. *Precision* is determined by the person separation index (number of levels of ability that can be distinguished in respondents) and person reliability (a measure of internal consistency). *Targeting* assesses whether person ability matches item difficulties. *Differential item functioning* assesses whether respondents from various subgroups with similar abilities respond differently to an item [17].

### 2.4 Responsiveness

Raw Catquest-9SF scores were converted to logit scores based on results of Analysis B (S2 Table). A higher raw score was translated to a more positive logit score. For each subject, each raw score was converted to logit for the item. Logit scores for individual items were summed and divided by 9 (total number of items), to determine the subject’s logit score on the questionnaire. Improvement in logit scores after surgery was assessed with a paired t-test. Mean and standard deviations (SD) are reported. p-values<0.05 were statistically significant.

### 2.5 Validation of Catquest-9SF subsets

Catquest-9SF is a relatively short questionnaire compared to other available tools. However, Sparrow et al. previously suggested that an even shorter questionnaire may be preferred [14]. In our previous validation in Ontario, we used Rasch analysis to explore the psychometric performance of 8-item, 7-item, and 5-item subsets of Catquest-9SF [17]. In all sub-analyses, reducing the number of items lowered precision. The best 8-item subset included items Ca,Cb,C1,C2,C3,C5,C6, and C7, and the best 5-item subset included items Cb,C1,C2,C3, and C7. The 8-item subset had excellent precision, distinguishing between low, medium, and high visual function, while the 5-item subset had adequate precision to distinguish only between low or high ability. In this study, Rasch analysis was performed on these two subsets.

Based on the above results, we would expect that shortening the questionnaire to less than five items would further reduce precision. However, an ultrashort questionnaire may provide unique benefits which may outweigh the limitation of reduced precision. For example, the Patient Health Questionnaire 2 (PHQ-2) is a two-item subset of PHQ-9, which is used for diagnosis of depression [34]. PHQ-2 has 95% sensitivity in diagnosis of depression and is commonly used in screening [35]. Thus, it is worthwhile to investigate the performance of an ultrashort subset of Catquest-9SF as it may help when administration of a full questionnaire is impractical.

We proposed a new 3-item subset of Catquest-9SF. Based on the best 5-item subset (Cb,C1,C2,C3,C7) from our previous analysis, the easiest item (C2-‘recognize faces’) and the most difficult item (Cb-‘satisfaction with vision’) were included so that respondents with extremes of visual function can be identified. For the third item, one of the remaining items–C1-‘read text in newspaper’, C3-‘see prices when shopping’, or C7-‘carry out a hobby’–could be chosen. In C7, ‘hobby’ could be interpreted differently based on respondents’ interests, as was the case in another study using this word to describe various activities [36]. In C1, not all respondents read newspapers, and the activity is not known to affect activities of daily living (ADL). C3 is related to grocery shopping, which is an instrumental ADL [37]. A limitation in the ability to shop is known to affect independence and quality of life [37]. Furthermore, vision impairment is associated with disability based on self-reported difficulty with ADLs, highlighting the potential importance of this item [38]. Thus, item C3 was chosen and Rasch analysis was re-run for the 3-item subset (Cb,C2,C3).

## 3 Results

### 3.1 Participants

Data was collected for 1786 patients. 1523 patients were included in Analysis A, with median age 72.0 (mean = 71.5,SD = 8.72), 518 (51.7%) female, and 696 (51.7%) completed high school or less. 934 subjects were included in Analysis B, with median age 72.0 (mean = 71.6,SD = 8.80), 492 (52.7%) female, and 387 (48.9%) completed high school or less (Table 1).

**Table 1:** Participant demographics.

	Pre-Op Only (Analysis A)	Combined Pre and Post-Op (Analysis B)
Total N	1523	934
**Age**	n = 985, missing = 538	n = 919, missing = 15
Median	72	72
Average (SD)	71.5 (8.72)	71.6 (8.80)
Range	39 to 100	39 to 100
**Gender**	n = 1001, missing = 522	n = 934, missing = 0
Female	518 (51.7%)	492 (52.7%)
Male	483 (48.3%)	442 (47.3%)
**Education**	n = 1346, missing = 177	N = 791, missing = 143
High school or less	696 (51.7%)	387 (48.9%)
More than high school	650 (48.3%)	404 (51.1%)
**Pre-op CDVA (better eye)**	n = 1523	n = 934
Median	0.3	0.3
Average	0.29 (0.22)	0.29 (0.21)
Range	-0.1 to 2.8	-0.1 to 2.8
**Pre-op CDVA (worse eye)**	n = 1523	n = 934
Median	0.48	0.4
Average (SD)	0.60 (0.47)	0.58 (0.46)
Range	0 to 3	0 to 3
**Post-op CDVA (better eye)**		N = 236, missing VA for at least one eye = 698
Median		0.1
Average (SD)		0.154 (0.12)
Range		-0.1 to 0.7
**Post-op CDVA (worse eye)**		N = 236, missing VA for at least one eye = 698
Median		0.18
Average (SD)		0.296 (0.35)
Range		-0.1 to 3
**Pre-Op Raw Catquest-9SF Score**		
n	n = 1296, missing at least one response = 227	n = 814, missing at least one response = 120
Average (SD)	18.83 (6.07)	18.44 (5.98)
**Post-Op Raw Catquest-9SF Score**		
n	n = 795, missing at least one response = 728	n = 795, missing at least one response = 139
Average (SD)	12.11 (4.25)	12.11 (4.25)

CDVA: Corrected distance visual acuity

### 3.2 Rasch analysis

In both Analysis A and B, Catquest-9SF demonstrated ordered category thresholds, acceptable fit statistics with one item misfitting, adequate precision, and unidimensionality. There was mistargeting, indicating that the items were relatively easy for respondent ability. The results for all criteria are outlined below.

#### 3.2.1 Threshold order

Category thresholds were ordered for all items (Analysis A: S2 Fig; Analysis B: S3 Fig).

#### 3.2.2 Item calibration and fit

Analysis A: Infit range was 0.85–1.38 and outfit range was 0.76–1.54 (Table 2). Item Cb-‘satisfaction with vision’ had an outfit value of 1.54. Analysis B: Infit range was 0.75–1.29 and outfit range was 0.74–1.51 (Table 2). Item Cb had an outfit value of 1.51. The acceptable range is 0.50–1.50, with values 1.5–2.0 being unproductive for measurement but not degrading.

**Table 2:** Rasch analysis results of Catquest-9SF.

		Pre-Operative Scores Only			Including Pre- and Post-Operative Scores		
Item	Question	Item Calibration* (SE)	Infit MNSQ	Outfit MNSQ	Item Calibration* (SE)	Infit MNSQ	Outfit MNSQ
Ca	Difficulties in daily life	-0.31 (0.04)	0.85	0.94	-0.27 (0.04)	0.75	0.82
Cb	Satisfaction with vision	-2.15 (0.04)	1.03	1.54	-1.77 (0.04)	1.20	1.51
C1	Read newspaper text	-0.51 (0.04)	1.09	1.06	-0.64 (0.04)	1.02	1.01
C2	Recognize faces	2.12 (0.06)	1.38	1.13	2.10 (0.06)	1.29	1.06
C3	See prices when shopping	-0.24 (0.04)	0.92	0.86	-0.43 (0.04)	0.86	0.85
C4	Walk on uneven ground	1.01 (0.05)	1.20	1.13	0.88 (0.05)	1.23	1.39
C5	Do needlework/handicraft	0.28 (0.05)	0.98	0.88	0.11 (0.05)	0.99	0.90
C6	Read text on television	-0.52 (0.04)	0.97	0.95	-0.34 (0.04)	0.99	0.97
C7	Carry out a hobby	0.32 (0.04)	0.85	0.76	0.35 (0.05)	0.85	0.74

*Measured in logits. Easier items that require lower visual function have positive values, while more difficult items requiring higher visual function have negative values.

#### 3.2.3 Unidimensionality

According to the principal component analysis of the residuals, the observed explained variance was close to the value expected if the data fit the Rasch model perfectly (Analysis A: observed = 61.3%,expected = 61.7%; Analysis B: observed = 60.4%,expected = 60.6%). The unexplained variance explained by the first contrast was 1.72 eigenvalue units in Analysis A and 1.75 in analysis B, which is less than 2.0 and therefore meets criteria for unidimensionality.

#### 3.2.4 Precision

Person separation index and person reliability were 2.49 and 0.86, respectively, in Analysis A and 2.01 and 0.80 in Analysis B, meaning that the questionnaire can discriminate respondents who have low, medium, and high abilities (minimum acceptable values are 2.00 and 0.80, respectively) [34]. Cronbach’s alpha was 0.90 in Analysis A and 0.92 in Analysis B, indicating excellent internal consistency (0.70 to <0.80 is acceptable, 0.80 to <0.90 is good, and 0.90 or higher is excellent) [14, 16].

#### 3.2.5 Targeting

The mean person location was -1.07 in Analysis A and -2.43 in Analysis B. Optimal value is between -1.00 and 1.00. Negative mistargeting means that respondents reported minimal difficulties with the questionnaire tasks.

#### 3.2.6 Differential Item Functioning (DIF)

Analysis A: There was no significant DIF for Catquest-9SF with respect to age. Small but statistically significant DIF (defined as DIF contrast between 0 and 0.5 and Rasch-Welch p<0.05) occurred as a function of gender for item Ca-‘difficulties in daily life’(DIF contrast = 0.27,p = 0.011,rated more difficult by men) and as a function of education level for C1-‘read newspaper text’(DIF contrast = 0.23,p = 0.0092,rated more difficult by those with education of high-school or less).

Analysis B: There was statistically significant DIF for some items with respect to age, gender, and pre-/post-operative status (Table 3). A detailed assessment of the pre-/post-operative DIF was performed using racked data, which focuses on the change in item difficulty, rather than person ability, from before surgery to after surgery (S3 Table). There was no DIF with respect to education level.

**Table 3:** Statistically significant DIF in pooled pre- and post-operative scores. Age groups were categorized as <65 years old and 65+ years old. DIF contrast <0.5 logits is considered small or absent, 0.50 to 1.0 logits is minimal, while >1.0 logits is notable.

Item	Variable	DIF contrast	p	Rated more difficult by
Ca (‘difficulties in daily life’)	Gender	0.21	0.018	Men
Cb (‘satisfaction with vision’)	Gender	0.22	0.0066	Men
	Pre/Post-op	0.81	<0.0001	Pre-op
C1 (‘read text in newspaper’)	Pre/Post-op	0.40	<0.0001	Post-op
C2 (‘recognize faces’)	Pre/Post-op	0.54	0.0010	Pre-op
C3 (‘see prices when shopping’)	Pre/Post-op	0.47	<0.0001	Post-op
C4 (‘walk on uneven ground’)	Age	0.28	0.029	65+ years old
C5 (‘do needlework/handicraft’)	Gender	0.21	0.026	Women
	Pre/Post-op	0.65	<0.0001	Post-op
C6 (‘read text on television’)	Age	0.24	0.032	65+ years old
	Pre/Post-op	0.39	0.0001	Pre-op

#### 3.2.7 Person-item map

**Fig 1:**
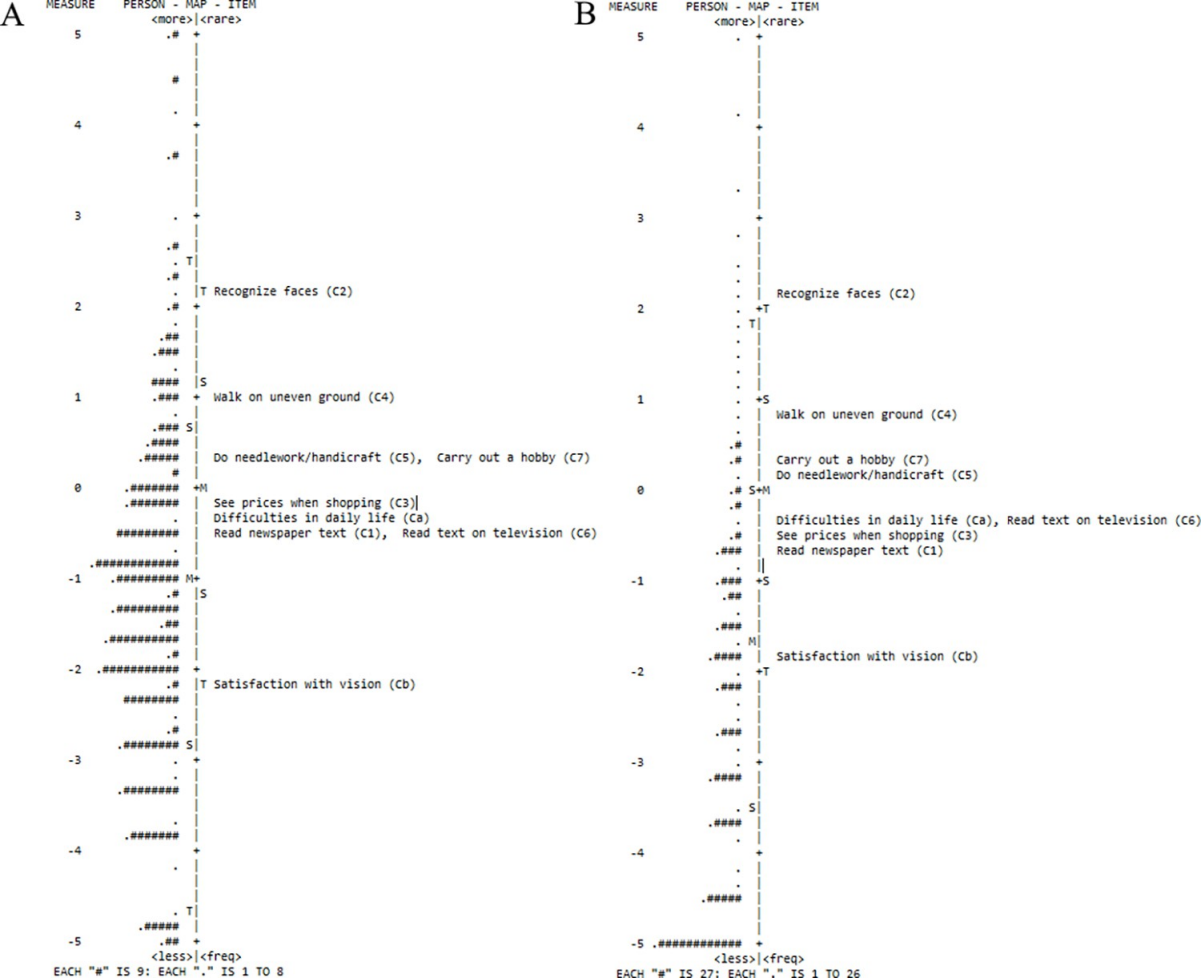
Person-item map for Catquest-9SF including A) pre-operative scores only and B) combined pre- and post-operative scores. The left side of the vertical line shows respondent data while the right side shows positioning of items. Respondents with higher visual function and items with higher difficulty are positioned at the bottom of the line. Each ‘#’ represents 2 respondents and each ‘.’ represents 1 respondent. M: mean, S:1 standard deviation, T: 2 standard deviations. The scale is in logits.

In both Analysis A and B, the easiest question was C2-‘recognize faces’, meaning that only respondents with very low visual function reported difficulty. The most difficult question was Cb-‘satisfaction with vision’, indicating that even respondents with high visual function reporting dissatisfaction. The person-item maps (Fig 1) show that C5-‘do needlework/handicraft’ and C7-‘carry out a hobby’ are on the same line. Items C3-‘see prices when shopping’,Ca-‘difficulties in daily life’,C1-‘read newspaper text’ and C6-‘read text on television’ are also at a similar position on the scale.

### 3.3 Responsiveness

**Fig 2:**
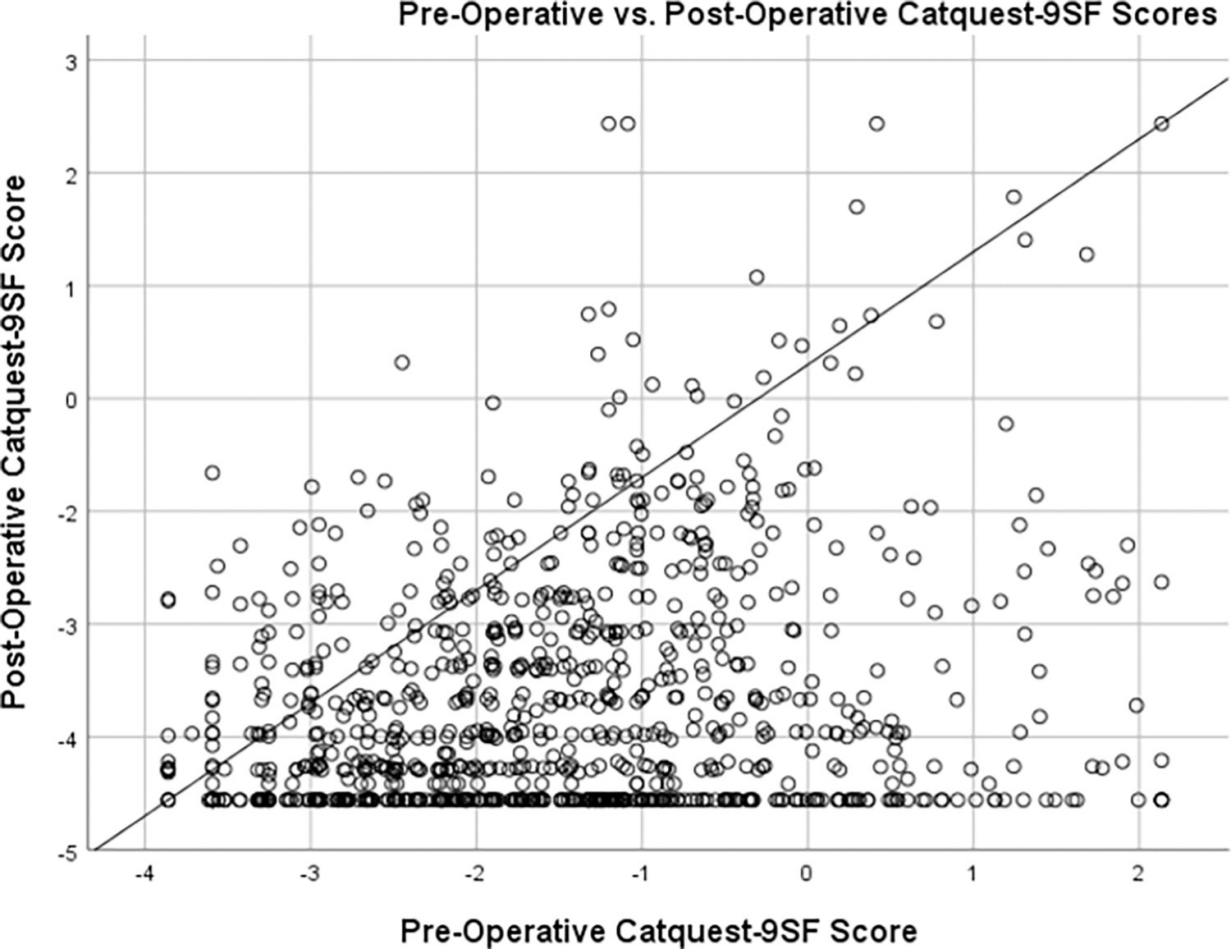
Scatterplot of pre-operative versus post-operative Catquest-9SF scores, in logits. Subjects who had improvement in visual function after cataract surgery fall under the diagonal line (a higher Catquest-9SF score represents poor visual function).

The raw scores were converted to logit scores and the mean logit score was used to determine questionnaire score pre- and post-operatively (S2 Table, S4 Fig). In most subjects, visual function based on total Catquest-9SF scores improved after cataract surgery, as expected (Fig 2). Of 934 subjects, 801 (85.8%) reported improvement, 8 (0.9%) reported no change, and 125 (13.4%) had decreased visual function. The mean pre-operative score was -1.70±1.3 logits, and the mean post-operative score was -3.17±1.1 logits. The improvement of 1.47 logits was statistically significant (p<0.001,paired 2-tailed t-test). All items became easier after surgery. Item Cb-‘satisfaction with vision’ had the largest change in item calibration and C5-‘do needlework/handicraft’ had the smallest.

An extended analysis was performed to further investigate why some subjects did not have improvement of Catquest-9SF scores after surgery (Table 4). Of 934 subjects, 809 had improvement or no change in Catquest-9SF scores after surgery compared to before surgery, and 125 subjects had worsening scores.

**Table 4:** Extended analysis on subjects who had improvement or worsening of Catquest-9SF scores from pre- to post-operatively. VA: Visual acuity. Those who had no change in score were grouped with those who had improvement in scores.

	N	Improved Catquest-9SF score	Worsened Catquest-9SF score	P (Mann Whitney U test)
Better Eye VA Pre-Op	934	0.30	0.30	0.102
Worse Eye VA Pre-Op	934	0.48	0.40	0.028
Better Eye VA Post-Op	236	0.10	0.14	0.307
Worse Eye VA Post-Op	236	0.18	0.18	0.705
Change in Better Eye VA	236	0.11	0.04	0.076
Change in Worse Eye VA	236	0.20	0.21	0.951
Catquest-9SF Scores Pre-Op	934	-1.62	-3.01	<0.001
Catquest-9SF Scores Post-Op	934	-3.76	-2.14	<0.001

There was a mild statistically significant correlation between change in vision in the better eye and change in Catquest-9SF scores (pre- to post-operatively; Pearson correlation = 0.19, p = 0.004, two-tailed significance). There was no correlation between change in vision in the worse eye and change in Catquest-9SF scores (p = 0.601, two-tailed significance).

Mann Whitney U test did not demonstrate a statistically significant difference in the change in Catquest-9SF scores (pre to post-op) as related to age, gender, and education level.

### 3.4 Validation of Catquest-9SF subsets

Assessment of questionnaire subsets using only pre-operative scores demonstrated acceptable psychometric properties for all shortened versions (Table 5). The 8-item subset had no misfitting items, adequate precision to distinguish between low, medium, and high visual function with person separation index (PSI) = 2.41, person reliability (PR) = 0.85, and acceptable targeting (-0.97). The 5-item and 3-item combinations had one misfitting item (Cb-‘satisfaction with vision’) and adequate precision to distinguish between low and high visual function (PSI = 1.94,PR = 0.79 for 5-item; PSI = 1.53,PR = 0.70 for 3-item; acceptable values are PSI>1.5 and PR>0.7, for this level of precision). There was mild mistargeting for both versions (-1.06 for 5-item, -1.23 for 3-item), indicating that the tasks described in the items were relatively easy for respondents to perform. There was no differential item functioning of magnitude above 0.50.

**Table 5:** Rasch analysis on subsets of Catquest-9SF items. Separate analyses presented for pre-operative scores only and combined pre- and post-operative scores (stacked data). *Presented as item: DIF contrast (p-value). PSI: person separation index, PR: person reliability.

	Catquest-9SF	8-Item Subset	5-Item Subset	3-Item Subset
Items Included	Ca, Cb, C1, C2, C3, C4, C5, C6, C7	Ca, Cb, C1, C2, C3, C5, C6, C7	Cb, C1, C2, C3, C7	Cb, C2, C3
Items Removed	None	C4	Ca, C4, C5, C6	Ca, C1, C4, C5, C6, C7
**Pre-Operative Scores Only**				
Ordered category thresholds?	Yes	Yes	Yes	Yes
Infit Range	0.85–1.38	0.87–1.44	0.85–1.35	0.92–1.13
Outfit Range	0.76–1.13 (Cb = 1.54)	0.78–1.49	0.80–1.12 (Cb = 1.72)	0.88–0.90 (Cb = 1.73)
Variance explained by measures, % (empirical calculation, expected)	61.3, 61.7	62.2, 62.6	67.3, 67.3	75.1, 74.4
Precision (PSI, PR)	2.49, 0.86	2.41, 0.85	1.94, 0.79	1.53, 0.70
Targeting	-1.07	-0.97	-1.06	-1.23
DIF age	No	No	No	No
DIF education*	C1: 0.23 (p = 0.01)	C1: 0.23 (p = 0.01)	C1: 0.22 (p = 0.02)	No
DIF sex*	Ca: 0.27 (p = 0.01)	Ca: 0.27 (p = 0.01)	No	No
Easiest item	C2	C2	C2	C2
Most difficult item	Cb	Cb	Cb	Cb
**Combined Pre- and Post-Operative Scores**				
Ordered category thresholds?	Yes	Yes	Yes	Yes
Infit Range	0.75–1.29	0.77–1.35	0.80–1.27	0.93–1.06
Outfit Range	0.74–1.39 (Cb = 1.51)	0.76–1.17 (Cb = 1.53)	0.79–1.19 (Cb = 1.64)	0.99–1.33
Variance explained by measures, % (empirical calculation, expected)	60.4, 60.6	61.3, 61.6	64.8, 64.9	71.2, 70.5
Precision (PSI, PR)	2.01, 0.80	2.01, 0.80	1.68, 0.74	1.38, 0.66
Targeting	-2.43	-2.40	-2.45	-2.69
DIF age*	C4: 0.28 (p = 0.03); C6: 0.24 (p = 0.03)	C6: 0.28 (p = 0.02)	No	No
DIF education	No	No	No	No
DIF sex*	Ca: 0.21 (p = 0.02); Cb: 0.22 (p = 0.01); C5: 0.21 (p = 0.03)	Ca: 0.21 (p = 0.02); Cb: 0.21 (p = 0.01); C5: 0.23 (p = 0.02)	Cb: 0.22 (p = 0.01)	C3: 0.20 (p = 0.04)
Easiest item	C2	C2	C2	C2
Most difficult item	Cb	Cb	Cb	Cb

Assessment of questionnaire subsets using both pre-operative and post-operative scores demonstrated acceptable psychometric properties for the 8-item and 5-item versions (Table 5). Item Cb misfit in both subsets with outfit values 1.53 (8-item) and 1.64 (5-item). The 8-item combination had adequate precision to discriminate between three levels of visual function (PSI = 2.01,PR = 0.80, acceptable values are PSI>2.0,PR>0.8), and the 5-item version had adequate precision to discriminate between two groups (PSI = 1.68,PR = 0.74, acceptable values are PSI>1.5,PR>0.7). The 3-item questionnaire had acceptable fit statistics with no misfitting items. Precision was inadequate, meaning that the questionnaire could not distinguish between at least two levels of visual function (PSI = 1.38,PR = 0.66). There was significant mistargeting in all three versions (-2.40,-2.45,-2.69 for the 8-item, 5-item, 3-item versions, respectively).

## 4 Discussion

Catquest-9SF demonstrated excellent Rasch-based psychometric properties in 12 world populations [12–16]. The questionnaire was previously validated in Peel Region, Ontario, Canada, using pre-operative scores only [17]. The current study assessed its performance in a large sample of pre- and post-operative cataract patients across 3 centers in Ontario. We show that Catquest-9SF has valid psychometric properties and is a suitable visual function questionnaire for use in Ontario. Implementation into routine care may aid in assessing appropriateness for cataract surgery and prioritization on waitlists. The questionnaire is also responsive to changes in visual function after cataract surgery, making it a potentially useful tool for assessment of surgical outcomes. Those who had improvement in Catquest-9SF scores from pre- to post-op had worse pre-operative visual acuity. Thus, subjects with worse visual acuity pre-operatively likely report greater improvement in their ability to perform daily activities when the vision improves, which is expected. There were no other variables that contributed to why a subject reported improvement or worsening of Catquest-9SF scores after surgery.

Catquest-9SF fulfilled all Rasch criteria. Precision was adequate to separate respondents based on low, medium, and high visual function. There was some mistargeting, meaning that the items were relatively easy for respondents. Mistargeting was greater when post-operative scores were included, which is expected because cataract surgery usually improves visual function.

Our Rasch analysis of Catquest-9SF showed similar results to the previous preoperative validation study in Ontario [17, S4 Table] and studies in other countries [39]. There is notable improvement with targeting in the current study (-1.07 pre-operatively), compared to the previous Ontario study (-1.43) [17]. Mistargeting was common in other studies (range -1.36 to -1.61) [16, 19, 21, 25, 39]. In all previous research, including the Ontario study, category thresholds were ordered, unidimensionality was confirmed, precision was adequate, and the person-item maps were similar.

In the previous Ontario study, all items demonstrated acceptable fit statistics. In this study, one item (Cb–‘satisfaction with vision’) had an outfit of 1.54 (maximum acceptable value = 1.50). In this case, high outfit may be because respondents with low visual function reported that they were satisfied with vision, or vice versa. In previous validation studies, misfitting items were removed from analysis. One study removed C5-‘do needlework/handicraft’ and another study removed C2-‘recognize faces’ and C4-‘walk on uneven ground’ [20, 24]. We did not remove the misfitting item because the outfit value was not degrading to the measurement. Furthermore, in a previous study where Catquest-9SF was shortened to 5 items, Cb was among the important questions to keep in the questionnaire [17].

There was no differential item functioning with respect to age, gender, or education level (DIF contrast>0.5). All items became easier after surgery, as expected. For some items, the improvements in scores were greater (Cb,C2,C6) and for others lower (C1,C3,C5) than expected based on the average improvement on all items. This shows that patients report different levels of improvement in visual function for different activities.

Shortened versions of the Catquest-9SF demonstrated promising results. The 8-item subset had excellent psychometric properties and performed better than the 9-item version in some areas. For example, pre-operatively, Catquest-9SF had one misfitting item and mild mistargeting, while the 8-item subset had excellent fit and targeting. In the analysis including pre- and post-operative scores, the 8-item and 9-item questionnaires performed similarly. Considering both analyses, the 8-item subset demonstrated better psychometric properties while also being shorter than Catquest-9SF.

The main limitation of the 5-item and 3-item versions is reduced precision compared to Catquest-9SF, and this is consistent with previous findings in Ontario [17]. The 5-item subset could not discriminate between three levels of ability like the 9-item version, but there was adequate precision to discriminate between two levels. This was the case in both the current study and the previous validation study in Ontario [17].

In analyzing only pre-operative scores, the 3-item subset performed similarly to Catquest-9SF except that it had adequate precision to discriminate between two, but not three, levels of visual function. Targeting was slightly reduced relative to Catquest-9SF. However, in the combined pre- and post-operative analysis, the 3-item version had unacceptable precision. Thus, the 3-item subset may be more suitable for analysis of only pre-operative visual function rather than combined pre-operative and post-operative data.

The main strengths of this study are the large sample size across three centers in different regions of Ontario and the availability of pre-operative and post-operative scores for assessment of responsiveness. One important limitation is that our pre- and post-operative scores come from the same subject. This violates an assumption in Rasch analysis which requires that all observations be independent [40]. One previous study changed the study design to prevent this violation [14], while all other studies stacked the pre-operative and post-operative scores into one dataset, which was our approach [12, 15, 19, 20, 22–25]. We report the results of the Rasch analysis on only pre-operative scores, where all observations are independent, to ensure that this did not inflate reliability and precision. Furthermore, due to our large sample size spanning several sites, there was missing data (Table 1). Also, since this is a pooled analysis, we do not have information on which eye (left or right) was operated on, so analysis was performed by categorizing visual acuity as ‘better’ or ‘worse’ eye.

In conclusion, Catquest-9SF demonstrated excellent psychometric properties and is a valid and reliable tool for measuring visual function before and after cataract surgery in Ontario. There is some mistargeting which indicates that the tasks are easy to perform, which is consistent with findings in other populations. Shortened variations of Catquest-9SF may be suitable for use, particularly pre-operatively. Future research should explore implementation of Catquest-9SF for clinical decision-making.

## Supporting information

S1 Fig Catquest-9SF questionnaire. (TIF)

S2 Fig Category probability curves for Catquest-9SF (including only pre-op scores). Including all 9 items.
(TIF)

S3 Fig Category probability curves for Catquest-9SF (including pre and post-op scores). Including all 9 items.
(TIF)

S4 Fig Distribution of pre-operative and post-operative Catquest-9SF logit scores. N = 934.
(TIF)

S1 Table Sensitivity analysis of demographic factors between sites. (DOCX)

S2 Table Conversion table between raw scores on Catquest-9SF to logit scores. Based on combined analysis of pre- and post-operative scores in 934 subjects. Raw score legend: 1 = No difficulty, 2 = Some difficulty, 3 = Great difficulty, 4 = Very great difficulty.
(DOCX)

S3 Table Assessment of differential item functioning for pre vs. post-operative groups. Data analyzed through racked approach. Total N = 934; 38 subjects were missing at least one item response for both pre- and post-operative Catquest-9SF; subjects with missing data were still accounted for according to the conversion table for logit scores. DIF: Differential item functioning. SE: Standard error.
(DOCX)

S4 Table Comparison of Rasch analysis results of current multi-center study in Ontario, Canada and previous single-center study in Ontario, Canada [17]. PSI: Person Separation Index, PR: Person Reliability.
(DOCX)

S1 Data (XLSX)
