# Supplementary figures and images for: Multi-center validation of Catquest-9SF visual function questionnaire in Ontario, Canada
Source: PLoS One. 2023 Jul 6;18(7):e0278863. doi: 10.1371/journal.pone.0278863 (PMC10325044; doi:10.1371/journal.pone.0278863)

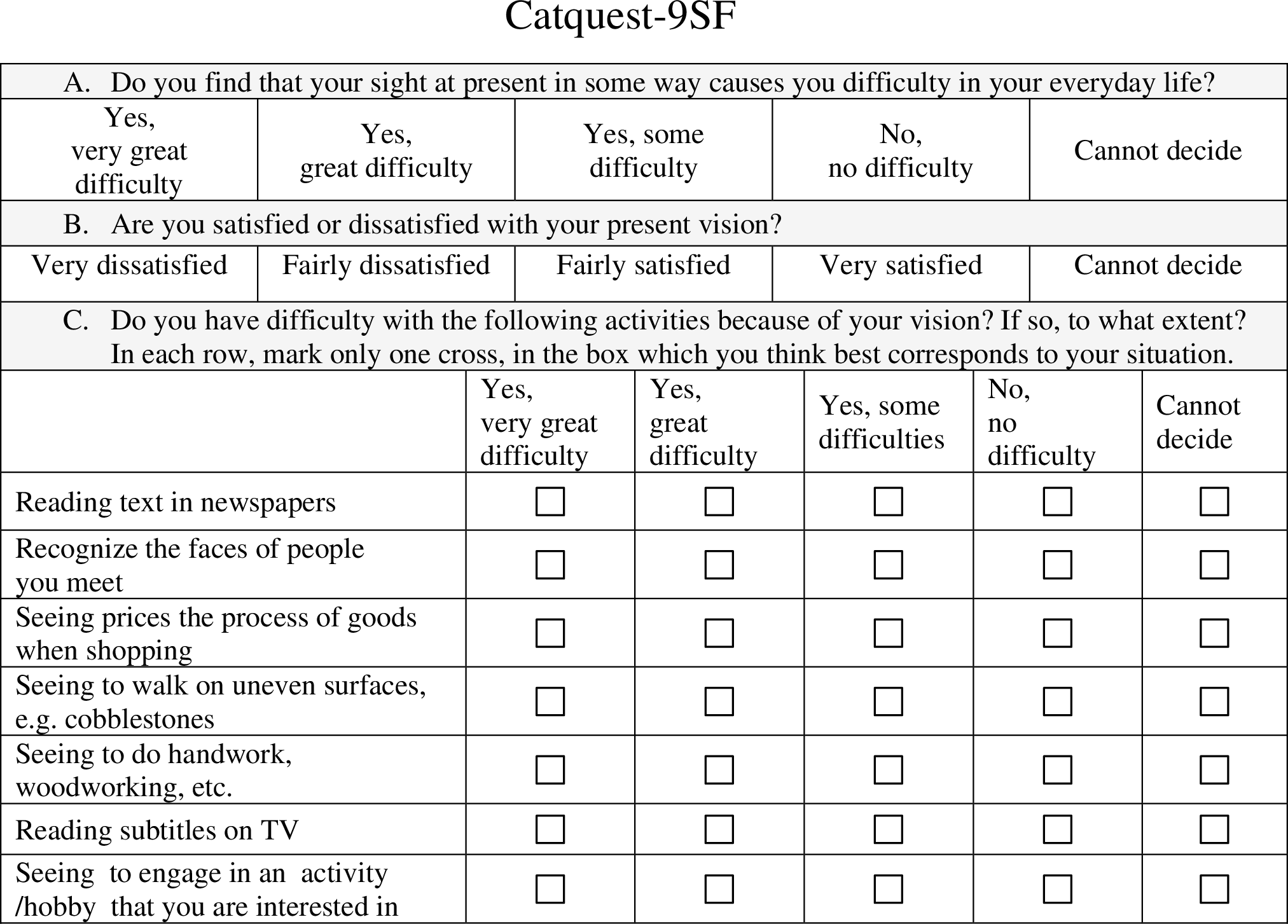

Supplement: S1 Fig — (TIF) [file pone.0278863.s001.tif]

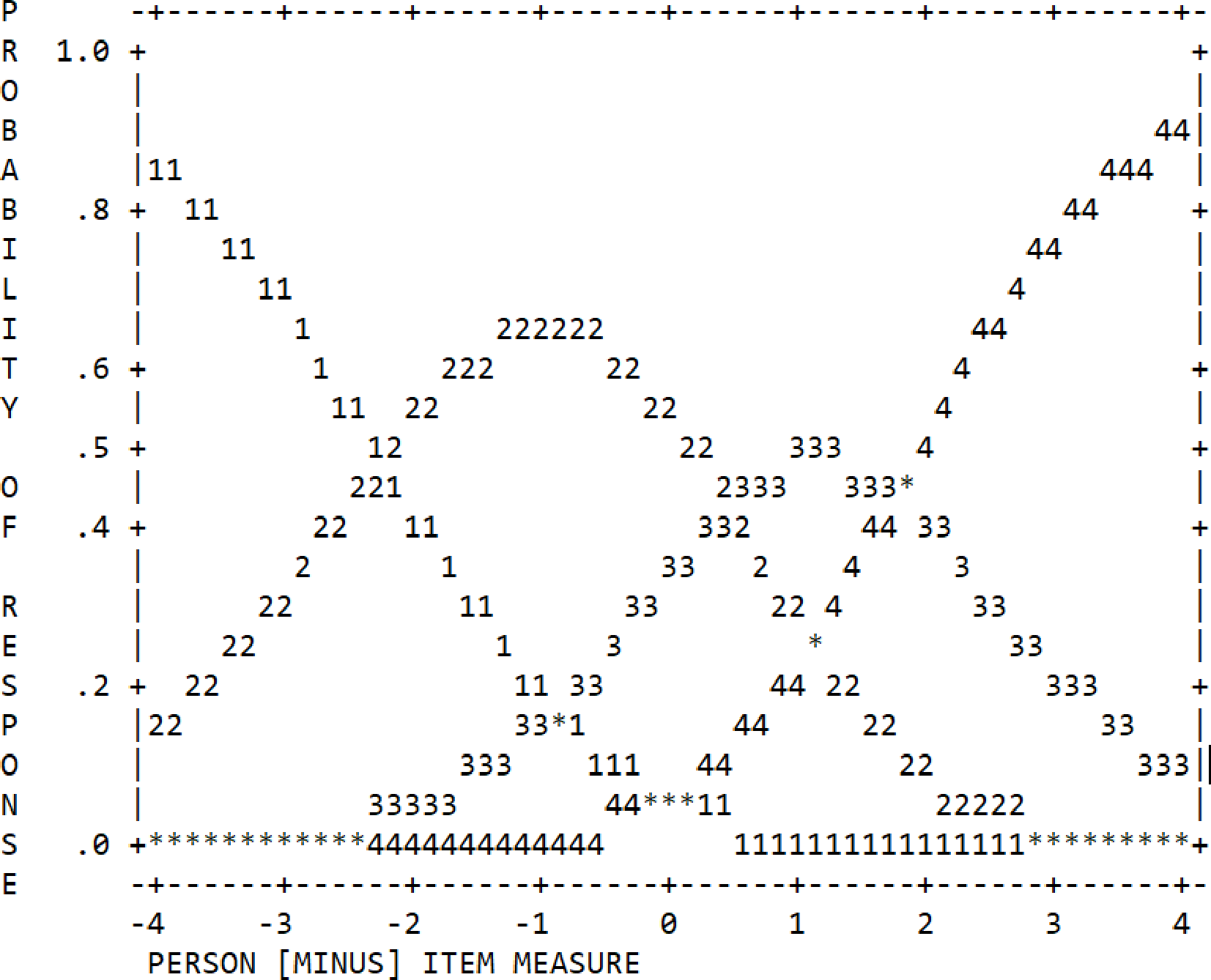

Supplement: S2 Fig — Including all 9 items. (TIF) [file pone.0278863.s002.tif]

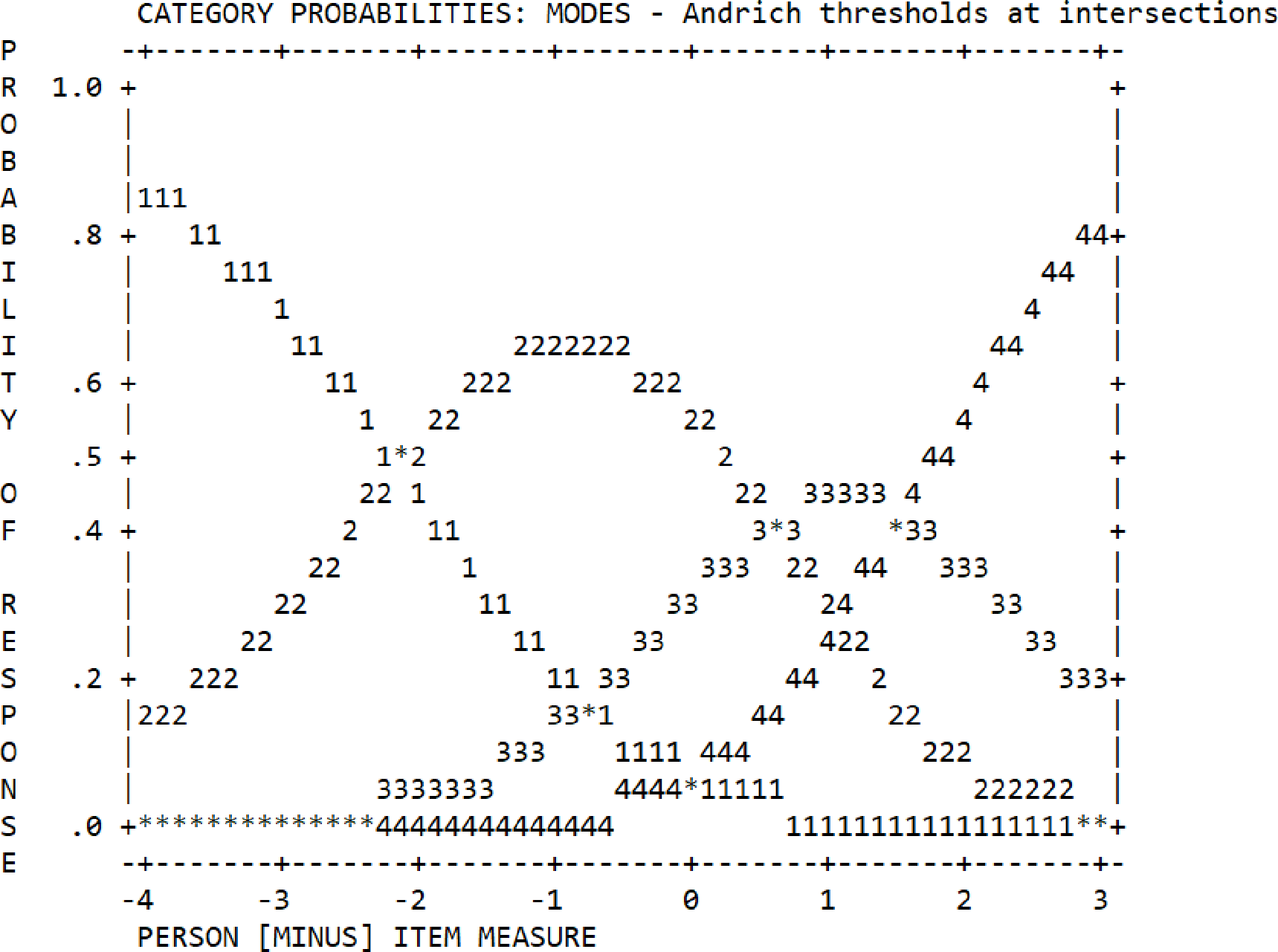

Supplement: S3 Fig — Including all 9 items. (TIF) [file pone.0278863.s003.tif]

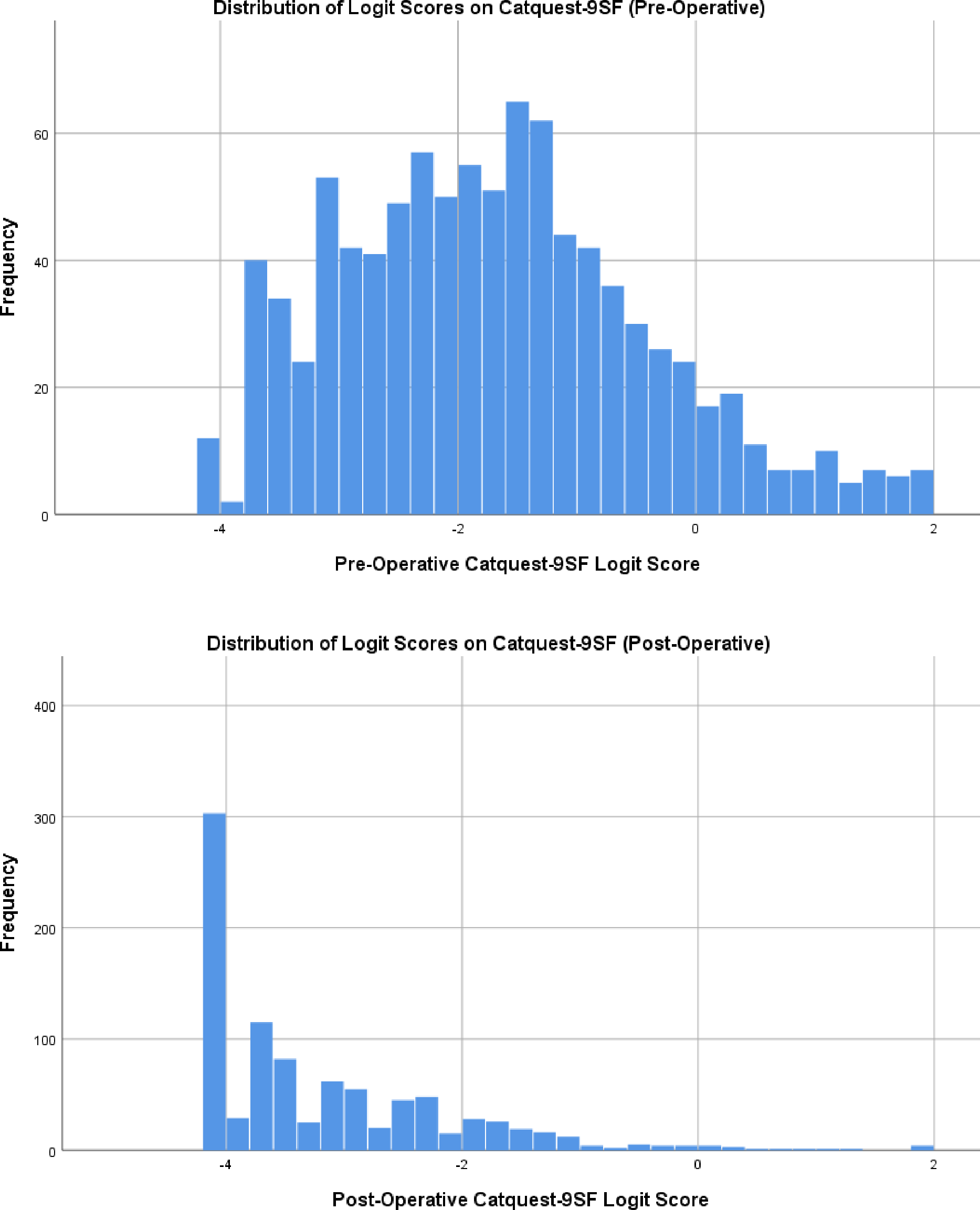

Supplement: S4 Fig — N = 934. (TIF) [file pone.0278863.s004.tif]
