# Supplementary material for: Multi-center validation of Catquest-9SF visual function questionnaire in Ontario, Canada
Source: PLoS One. 2023 Jul 6;18(7):e0278863. doi: 10.1371/journal.pone.0278863 (PMC10325044; doi:10.1371/journal.pone.0278863)
Supplement: S1 Table — (DOCX) [file pone.0278863.s005.docx]

**S1 Table: Sensitivity analysis of demographic factors between sites.**

|  | Site 1 | Site 2 | Site 3 |
| --- | --- | --- | --- |
| Total n | 308 | 1048 | 167 |
| **Age** | | | |
| Missing data | 0 | 538 | 0 |
| Median | 70.0 | 74.0 | 71.0 |
| Mean (SD) | 69.2 (8.2) | 73.4 (8.7) | 70.1 (8.4) |
| Range | 42-84 | 43-100 | 39-88 |
| **Gender** | | | |
| Missing data | 0 | 522 | 0 |
| Females | 175 (56.8%) | 271 (51.5%) | 72 (43.1%) |
| Males | 133 (43.2%) | 255 (48.5%) | 95 (56.9%) |
| **Education** | | | |
| Missing data | 0 | 10 | 167 |
| High school or less | 131 (42.5%) | 565 (54.4%) | N/A |
| More than high school | 177 (57.5%) | 473 (45.6%) | N/A |
| **Pre-Op BCVA (Better Eye)** (no missing data) | | | |
| Median | 0.30 | 0.30 | 0.18 |
| Mean (SD) | 0.26 (0.2) | 0.31 (0.21) | 0.24 (0.27) |
| Range | 0-2.0 | 0-2.0 | -0.1-2.8 |
| **Pre-Op BCVA (Worse Eye)** (no missing data) | | | |
| Median | 0.48 | 0.48 | 0.40 |
| Mean (SD) | 0.64 (0.52) | 0.61 (0.46) | 0.50 (0.43) |
| Range | 0.1-3.0 | 0-3.0 | 0-2.8 |
