# Supplementary material for: Multi-center validation of Catquest-9SF visual function questionnaire in Ontario, Canada
Source: PLoS One. 2023 Jul 6;18(7):e0278863. doi: 10.1371/journal.pone.0278863 (PMC10325044; doi:10.1371/journal.pone.0278863)
Supplement: S2 Table — Based on combined analysis of pre- and post-operative scores in 934 subjects. Raw score legend: 1 = No difficulty, 2 = Some difficulty, 3 = Great difficulty, 4 = Very great difficulty. (DOCX) [file pone.0278863.s006.docx]

**S2 Table: Conversion table between raw scores on Catquest-9SF to logit scores.** Based on combined analysis of pre- and post-operative scores in 934 subjects. Raw score legend: 1=No difficulty, 2=Some difficulty, 3=Great difficulty, 4=Very great difficulty

| Raw Score: | | 1 | 2 | 3 | 4 | Missing |
| --- | --- | --- | --- | --- | --- | --- |
| **Ca** | Difficulties in daily life | -4.53 | -1.47 | 0.43 | 2.28 | -2.32 |
| **Cb** | Satisfaction with vision | -4.86 | -2.46 | -0.94 | 0.43 | -1.70 |
| **C1** | Read newspaper text | -4.42 | -1.69 | -0.08 | 1.18 | -2.74 |
| **C2** | Recognize faces | -3.05 | -0.43 | 1.15 | 3.28 | -1.46 |
| **C3** | See prices when shopping | -4.27 | -1.59 | -0.03 | 1.63 | -1.86 |
| **C4** | Walk on uneven ground | -3.49 | -1.03 | 0.36 | 2.69 | -1.31 |
| **C5** | Do needlework/handicraft | -3.92 | -1.28 | 0.20 | 1.97 | -2.63 |
| **C6** | Read text on television | -4.12 | -1.58 | -0.09 | 1.65 | -1.93 |
| **C7** | Carry out a hobby | -3.88 | -1.08 | 0.39 | 2.31 | -1.91 |
