# Supplementary material for: Multi-center validation of Catquest-9SF visual function questionnaire in Ontario, Canada
Source: PLoS One. 2023 Jul 6;18(7):e0278863. doi: 10.1371/journal.pone.0278863 (PMC10325044; doi:10.1371/journal.pone.0278863)
Supplement: S3 Table — Data analyzed through racked approach. Total N = 934; 38 subjects were missing at least one item response for both pre- and post-operative Catquest-9SF; subjects with missing data were still accounted for according to the conversion table for logit scores. DIF: Differential item functioning. SE: Standard error. (DOCX) [file pone.0278863.s007.docx]

**S3 Table: Assessment of differential item functioning for pre vs. post-operative groups**. Data analyzed through racked approach. Total N = 934; 38 subjects were missing at least one item response for both pre- and post-operative Catquest-9SF; subjects with missing data were still accounted for according to the conversion table for logit scores. DIF: Differential item functioning. SE: Standard error.

| Item | Question | DIF result: group experiencing more difficulty with item | Pre-Operative | | | Post-Operative | | | Pre vs. Post-Operative | |
| --- | --- | --- | --- | --- | --- | --- | --- | --- | --- | --- |
|  |  |  | Average logit score (# of missing data) | Item calibration† (SE) | Relative to mean item calibration (0.82) | Average logit score (# of missing data) | Item calibration† (SE) | Relative to mean item calibration (2.66) | Difference in item calibration | Difference in average logit score |
| Ca | Difficulties in daily life | No DIF | -1.44 (14) | 0.62 | 0.2 | -3.43 (19) | 2.49 | 0.17 | 1.87 | 1.99 |
| Cb | Satisfaction with vision | Pre-Op | -1.24 (13) | -0.68 | 1.5 | -3.62 (15) | 1.83 | 0.83 | 2.51 | 2.38 |
| C1 | Read newspaper text | Post-Op | -1.69 (3) | 0.46 | 0.36 | -3.18 (9) | 2.03 | 0.63 | 1.57 | 1.49 |
| C2 | Recognize faces | Pre-Op | -2.02 (5) | 2.27 | -1.45 | -2.85 (2) | 4.44 | -1.78 | 2.17 | 0.83 |
| C3 | See prices when shopping | Post-Op | -1.73 (6) | 0.63 | 0.19 | -3.13 (10) | 2.14 | 0.52 | 1.51 | 1.40 |
| C4 | Walk on uneven ground | No DIF | -1.90 (13) | 1.46 | -0.64 | -2.97 (9) | 3.23 | -0.57 | 1.77 | 1.07 |
| C5 | Do needlework/handicraft | Post-Op | -1.93 (58) | 1.06 | -0.24 | -2.93 (72) | 2.40 | 0.26 | 1.34 | 1.00 |
| C6 | Read text on television | Pre-Op | -1.56 (7) | 0.50 | 0.32 | -3.30 (9) | 2.59 | 0.07 | 2.09 | 1.74 |
| C7 | Carry out a hobby | No DIF | -1.77 (18) | 1.10 | -0.28 | -3.10 (16) | 2.82 | -0.16 | 1.72 | 1.33 |
|  | **Mean** |  |  | 0.82 |  |  | 2.66 |  | 1.84 | 1.47 |
