# Supplementary material for: Multi-center validation of Catquest-9SF visual function questionnaire in Ontario, Canada
Source: PLoS One. 2023 Jul 6;18(7):e0278863. doi: 10.1371/journal.pone.0278863 (PMC10325044; doi:10.1371/journal.pone.0278863)
Supplement: S4 Table — PSI: Person Separation Index, PR: Person Reliability. (DOCX) [file pone.0278863.s008.docx]

**S4 Table: Comparison of Rasch analysis results of current multi-center study in Ontario, Canada and previous single-center study in Ontario, Canada [17].**

PSI: Person Separation Index, PR: Person Reliability

|  |  | Previous Study | Current Study | |
| --- | --- | --- | --- | --- |
|  | Acceptable values | Pre-op only | Pre-op only | Pre- and Post-op |
| N |  | 313 | 1523 | 934 |
| Ordered thresholds | Yes | Yes | Yes | Yes |
| Infit range | Within 0.50 – 1.50 | 0.75-1.35 | 0.85-1.38 | 0.75-1.29 |
| Outfit range | Within 0.50 – 1.50 | 0.83-1.36 | 0.76-1.54 | 0.74-1.51 |
| Unidimensionality: variance unexplained by measures (observed, expected) | <50% and comparable between observed and expected values | 41.7%, 41.0% | 38.7%, 38.3% | 39.6%, 39.4% |
| Unexplained variance explained by the first contrast | <2.00 | 1.70 | 1.72 | 1.75 |
| Precision: PSI, PR | >=2.00, >=0.80 | 2.09, 0.81 | 2.49, 0.86 | 2.01, 0.80 |
| Targeting | Within -1.0 to 1.0 | -1.43 | -1.07 | -2.43 |
